# Effectiveness and safety of ultrasound-guided percutaneous microwave ablation for hepatic alveolar echinococcosis

**DOI:** 10.1186/s12880-022-00752-2

**Published:** 2022-02-12

**Authors:** Xu Deng, Jing-jing Wang, Zhi-xin Wang, Hai-ning Fan, Hai-jiu Wang, Han-sheng Huang, Kai-qaing Wang, Xiao-zhou Yang, Jun-wei Han, Yangdan Cairang

**Affiliations:** 1 Department of Hepatopancreatobiliary Surgery, The Affiliated Hospital of Qinghai University, Xining, China; 2 Qinghai Province Key Laboratory of Hydatid Disease Research, Xining, China

**Keywords:** Alveolar echinococcosis, Multilocular echinococcosis, Hepatic alveolar echinococcosis, Microwave ablation

## Abstract

**Background:**

Microwave ablation (MWA) is a popular therapy for liver malignant tumor in recent years. Few studies have been conducted on its use in the treatment of hepatic alveolar echinococcosis (HAE). The study aims to evaluate the efficacy and safety of MWA in the treatment of HAE.

**Methods:**

This study analyzed the data of 45 patients (mean age, 38 ± 2 years; 24 males) diagnosed with HAE and underwent MWA treatment between June 2014 to December 2019. The patients after MWA were examined by CT or MRI [follow-up: 32 months (IQR 23–48.5)] to determine whether the lesions were relapsed and to evaluate the therapeutic effect of MWA. The safety of MWA was evaluated by monitoring postoperative complications. Clinical data, such as patient demographics, imaging features of the lesions, relevant findings of laboratory tests before and after ablation, and information related to ablation, were collected and analyzed. Paired-sample t tests and paired-sample Wilcoxon signed-rank tests were used to compare relevant laboratory indicators before and after MWA.

**Results:**

MWA was applied to 57 HAE lesions in 45 patients. The median size of lesions was 3.42 cm (IQR2.85–4.41). The rate of complete ablation was 100% (57/57). The median follow-up time was 32 months (IQR 23–48.5). The recurrence rate was 13% (6/45), and the median time of recurrence was 22 months. The rate of minor complications was 11.1% (5/45), and there were no major complications and deaths. Compared to preoperative, ALB, RBC, HBG, and PLT were decreased (*p* < 0.001); ALT, TB, DB, and WBC were increased (*p* < 0.001); and no statistically difference in PT, APTT, and INR (*p* > 0.05).

**Conclusions:**

MWA might be a safe and effective way to cure HAE. Meanwhile, it provides a new option and a new way of thinking about treatment for patients with HAE.

## Background

Hepatic Alveolar Echinococcosis (HAE) is a zoonotic disease caused by the larvae of Echinococcus multilocularis. In China, Qinghai Province is considered to be endemic to this parasite, as well as Sichuan Province, Gansu Province, and the autonomous regions of Xinjiang and Tibet [1]. HAE is also called “parasitic cancer” because of its invasiveness, which is similar to the biological characteristics of a malignant tumor [2]. As far as we know, HAE in China and Europe does not differ much in terms of clinical symptoms. Clinical symptoms vary with the degree of organ invasion involved. Given that HAE mainly invades the liver, the symptoms are similar to the liver cancer. Most patients seek treatment in the hospital for symptoms such as abdominal pain, jaundice, and/or weight loss [3]. Without timely diagnosis and therapy, the prognosis is worse, and approximately 95% of patients will die [4]. Therefore, aggressive therapy is often necessary to eradicate the totality of the parasites.

At present, radically surgical resection combined with antiparasitic drug treatment has been considered the first choice for the treatment of HAE [4]. However, radical resection has more trauma and more postoperative complications than MWA in small lesions of liver cancer [5]. As we all know, MWA causes coagulative necrosis of the lesion tissue by generating high temperature around the lesion and has achieved good therapeutic results in the treatment of liver malignant tumor [6]. Therefore, MWA may be a good choice for the treatment of HAE.

To date, there are few reports about MWA for the treatment of HAE [7, 8]. The present study aimed to analyze the effectiveness and safety of MWA in the treatment of HAE.

## Methods

### Patient selection

This study was approved by the Medical Ethics Committee of the First Affiliated Hospital of Xinjiang Medical University and the Affiliated Hospital of Qinghai University (20190531-03), and was conducted according to the ethical guidelines of the Helsinki Declaration. Because this study was retrospective, we waived the requirement for written informed consent.

From June 2014 to December 2019, we collected the clinical information from 45 patients who were diagnosed with HAE in our institution. In a prior study, we reported on 17 patients who were included in this study [7]. The previous study preliminary documented the safety and effectiveness of MWA, whereas this study expands on this by having a larger patient number and a longer follow-up period.

Inclusion criteria were: (a) a diagnosis of HAE based on clinical presentation, epidemiological data, imaging, serology indexes, histopathology, and other items according to 2010 WHO expert consensus [2]; (b) lesions more than 2 cm from the first, second and third hepatic hilum without invasion of the bold ducts and vessels and without distant metastases; (c) no patients with severe coagulation dysfunction and hematological disorders; (d) Child–Pugh grade A or B. Since the diagnosis of HAE currently relies mainly on imaging, we have listed the imaging features of the main HAE lesions in the context of the current literature as follows: (a) US: irregularly contoured mass lesion with mixed heterogeneous echogenicity; (b) non-enhanced CT: masses with irregular borders, heterogeneous internal structure, and multiple distributed foci of calcification; (c) enhanced CT: No significant enhancement within the lesion; (d) MRI: masses with infiltrative features, irregular borders, internal heterogeneity, and a central area of necrosis; (e) DWI: restricted diffusion [9]. At the same time, we performed clinical and imaging staging based on their images [10, 11].

**Fig. 1:**
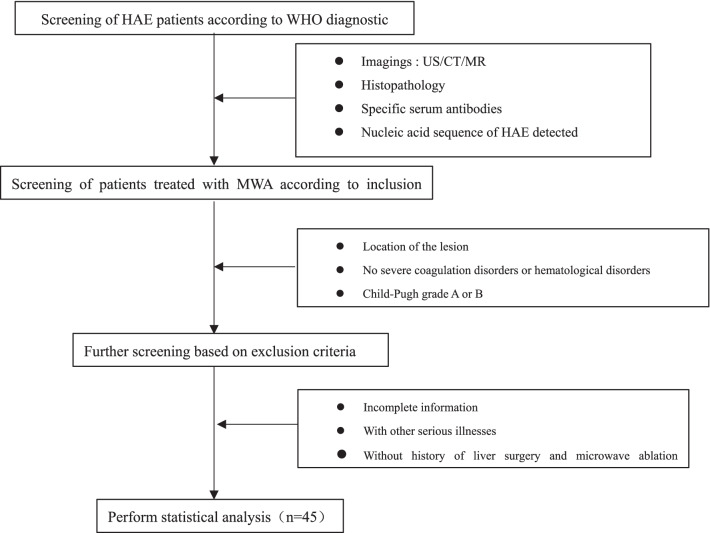
Research design diagram

The exclusion criteria were (a) patients whose basic treatment information was incomplete or who did not have follow-up data; (b) patients with severe cardiopulmonary dysfunction, or other contraindications to surgery; (c) Without history of liver surgery and microwave ablation treatment (Fig. 1).

### MWA procedure

The patient was treated with MWA by one of the authors(Y.C, 7 years). After the location of the lesion is determined by a ultrasonography (PHILIPS, CX50, M7 Series; the ultrasound probe model, 3C5s), a 15-gauge cooled antenna, and a 2.45 GHz generator with a power from 0 to 100 W (Nanjing Kang You Medical Technology Co, Ltd) were used to treat HAE. According to our experience, single-needle puncture single-point ablation is used for lesions within 3 cm. For lesions 3–5 cm, single-needle puncture multi-point ablation could be used. However, for lesions larger than 5.0 cm, complete ablation was achieved with two or more needles. The energy output and duration required for ablation were dependent on the location and size of the lesion, and the manufacturer's instructions. During the ablation process, a growing hyperechoic area was observed on US, indicating that the lesion was carbonizing [7]. The ablation stopped, when the hyperechoic area completely covered the lesion and at least 0.5 cm of normal liver parenchyma around the lesion. When withdraw the antenna, the needle track was ablated to prevent lesion seeding as well as control the bleeding. If a patient had multiple lesions, the same method was used. All complications related to MWA were recorded according to the Clavien–Dindo classification system.

### Assessment of clinical efficacy and safety

Imaging examinations such as CT or MRI and related laboratory tests were completed within 24 h after MWA [12]. Complete ablation was defined as the absence of any enhancing tissue at the ablation site at multiphase contrast-enhanced CT or MRI [5]. And it was valued by a chief (HL. Li, 23 years) or associate chief radiologist (L. Meng, 19 years) with many years of experience in abdominal imaging and familiarity with ablation techniques. For patients with incomplete ablation, we can choose to perform a second ablation or radical surgical resection when conditions permit, or regularly follow-up to monitor the progress of HAE. Recurrence was defined as the appearance of new lesions around the ablation zone with imaging characteristics of HAE. Distant metastasis was defined as any HAE lesions that appeared outside the liver. Postoperative complications were graded according to the Clavien–Dindo classification [13].

### Clinical data collection and follow-up

The clinical data of all patients were collected, included patient demographics (age, gender, etc.), clinical symptoms, relevant results of laboratory examinations before and after ablation, information related to ablation and imaging features of the lesion (size, location, number). Follow-up was performed once in the first and third months after discharge, and once every 3–6 months thereafter until the end of follow-up period. At each follow-up visit, liver function examination and abdominal imaging examination were performed in the outpatient department.

### Statistical analysis

Statistical analysis was performed with SPSS 26.0 (IBM Corp., Armonk, NY, USA) Categorical variables were expressed as numbers and summarized as the percentage of the total group. The Shapiro–Wilk test was used to determine whether the continuous variables conformed to the normality distribution. Normally distributed continuous variables were expressed as the means ± SD and were analyzed using the paired t-test. Nonnormally distributed continuous variables were expressed as medians and interquartile ranges (IQR) and were analyzed using the paired Wilcoxon-test. A P value less than 0.05 was considered statistically significant.

## Results

### Patient demographics and clinical characteristics

In our study, the MWA treatment response and complication rates of 45 patients (mean age, 38 ± 2 years; mean Body Mass Index, 22.9  ± 0.6 kg/m^2^; 24 males and 21 females;) with 57 HAE lesions were evaluated. The baseline clinical characteristics and the information about lesions and MWA were showed in Table 1. 45 patients underwent PNM staging and 57 lesions underwent calcification classification and Kodama classification (Table 2). Before MWA, symptoms appeared in 55.6% (25/45) of patients, including nonspecific abdominal pain, abdominal distension, and acid reflux. The remaining 44.4% (20/45) of patients were admitted to the hospital for treatment because of suspected HAE during routine medical examinations. Only two patients had a Child–Pugh classification of B, and the remaining patients has a Child–Pugh classification of A. There were also 13.3% (6/45) of patients with HBV. In addition, the median size of MWA-treated lesions was 3.42 cm (IQR 2.85–4.41). In our data, only 11.1% (5/57) of the lesions were larger than 5 cm in diameter, and 20% (9/45) of patients had two and more lesions. And most lesions were found in segments IV (24.6%) and VIII (21.1%) of the liver, while segments VI (15.8%), III (14.0%) and II (10.5%) were also common locations in the liver. There were 50.9% (29/57) of lesions located in the left liver and 49.1% (28/57) of lesions in the right liver. Ablation protocol and clinical results are summarized in Tables 3 and 4.

**Table 1:** Baseline clinical characteristics of patients with HAE at the time of MWA

Characteristic	Value
Mean age (year)^a^	38 ± 2
Mean body mass index (kg/m^2^)^a^	22.9 ± 0.6
Sex	
Male	24 (53.3)
Female	21 (46.7)
Clinical symptom	
Yes	25 (55.6)
No	20 (44.4)
Child–Pugh grade	
Child A	43 (95.6)
Child B	2 (4.4)
HBV	
Yes	6 (13.3)
No	39 (86.7)
*Nodules*	
Maximum diameter of nodules (cm)^b^	3.42 (2.85–4.41)
< 3 cm	19 (33.3)
3–5 cm	33 (57.9)
> 5 cm	5 (8.8)
Number of nodules	
1	36 (80.0)
2	6 (13.3)
3	3 (6.7)
Segmental location	
I	1 (1.8)
II	6 (10.5)
III	8 (14.0)
IV	14 (24.6)
V	4 (7.0)
VI	9 (15.8)
VII	3 (5.3)
VIII	12 (21.1)
Right hepatic lobe	28 (49.1)
Left hepatic lobe	29 (50.9)

Except where indicated, data are raw data, with percentages in parentheses

^a^Data are means ± standard deviation

^b^Data are median, with IQR in parentheses

**Table 2:** The classification of AE on the basis of PNM, CT calcification and Kodama

	Value
*PNM*	
P1N0M0	43 (95.56%)
P2N0M0	1 (2.22%)
P3N0M0	1 (2.22%)
*Pattern of calcification*	
Without calcifications	10 (17.54%)
With feathery calcifications	18 (31.58%)
With focal calcifications	6 (10.53%)
With diffuse calcifications	11 (19.30%)
With calcifications primarily at the edge	9 (15.79%)
With a central calcification	3 (5.26%)
*Kodama classification*	
Type 1	15 (26.32%)
Type 2	5 (8.77%)
Type 3	30 (52.63%)
Type 4	7 (12.28%)
Type 5	0 (0%)

A total of 57 lesions were identified in 45 patients, of which 45 patients underwent PNM staging and 57 lesions underwent calcification classification and Kodama classification

**Table 3:** Ablation protocol and clinical results

Ablation protocol	Value
Ablation time for nodule (min)^a^	5.0 (4.0–7.5)
Complete ablation	57 (100)
Complications (Clavien–Dindo)	
Minor (I–II)	5(11.1)
Post-ablation syndrome	1 (20)
Hypoproteinemia	1 (20)
Asymptomatic pleural effusions	3 (60)
Major (III_a_–IV_b_)	0
Mortality (V)	0
Follow-up (month)^a^	32 (23–48.5)
Recurrences/disease progressions	6 (13)
Hospital stay (day)^a^	5 (3–6)

Except where indicated, data are raw data, with percentages in parentheses

^a^Data are median, with IQR in parentheses

**Table 4:** The laboratory examinations before and after MWA

Laboratory Test	Before MWA	After MWA	*P* value※
ALT (U/L)^b^	27.00 (17.00–56.00)	130.00 (89.50–207.00)	0.000
TB (µmol/L)^b^	9.00 (6.70–12.65)	15.80 (11.50–26.90)	0.000
DB (µmol/L)^b^	3.50 (2.45–5.40)	5.20 (4.40–8.10)	0.000
ALB (g/L)^a^	40.18 ± 0.6	36.06 ± 0.51	0.000
WBC (10^9^/L)^a^	6.54 ± 0.28	9.97 ± 0.60	0.000
RBC (10^12^/L)^a^	4.93 ± 0.10	4.70 ± 0.10	0.000
HBG (g/L)^a^	148.93 ± 3.26	141.56 ± 3.32	0.000
PLT (10^9^/L)^a^	255.73 ± 15.17	220.84 ± 12.10	0.000
PT (s)^b^	11.10 (10.30–12.15)	11.60 (10.70–12.40)	0.053
APTT (s)^a^	30.80 ± 0.80	29.78 ± 0.83	0.072
INR^b^	0.92 (0.86–1.01)	0.96 (0.87–1.03)	0.102

※*P* < 0.05 was considered statistically significant

^a^Data are means ± standard deviation, analyzed using the paired t-test

^b^Data are median, with IQR in parentheses, analyzed using the paired Wilcoxon-test

### The effectiveness evaluation of MWA

**Fig. 2:**
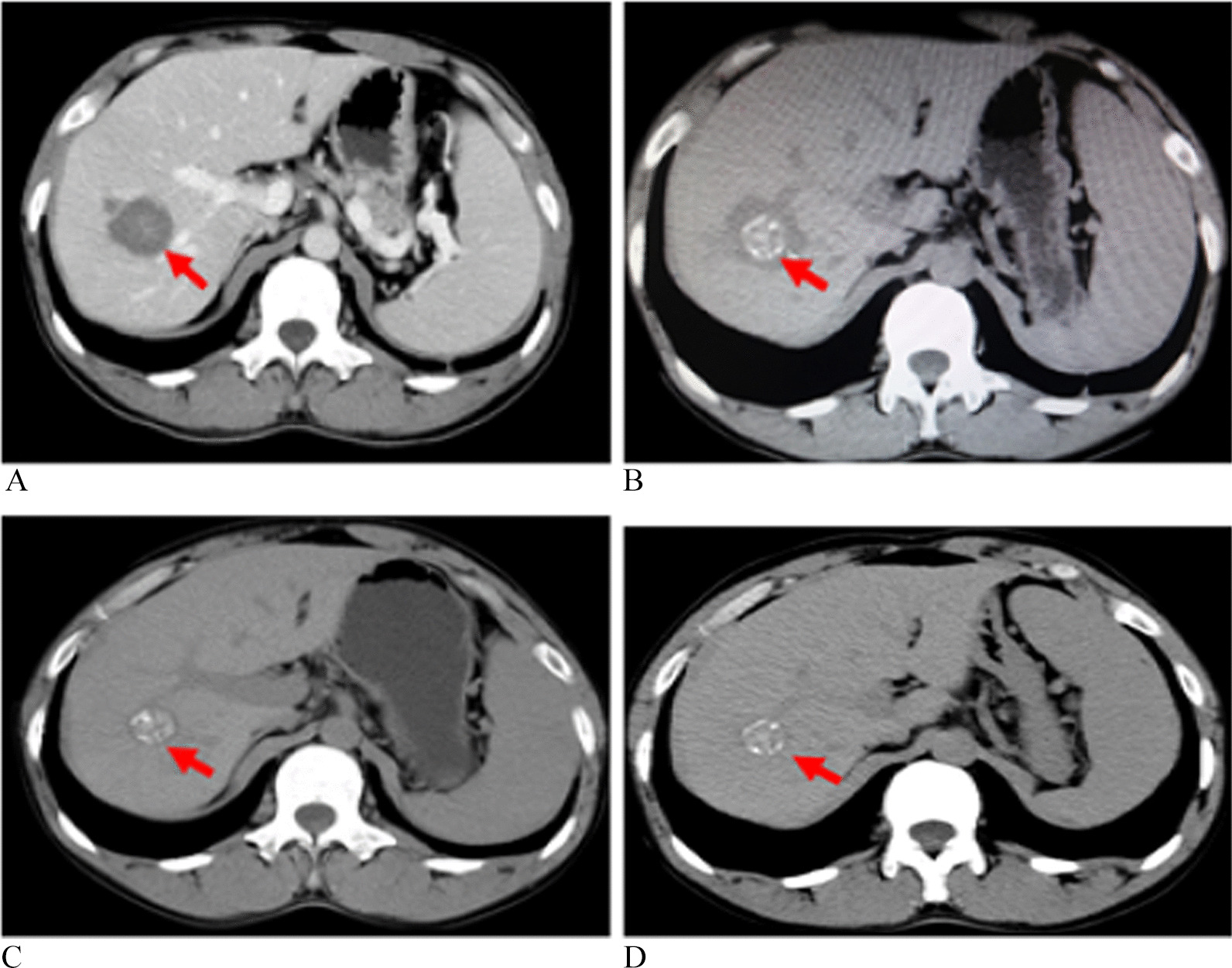
Images showing the lesions before and 1, 6, 12 months after MWA in a 36-year-old male with HAE. **A** Enhanced CT was performed before ablation. **B** CT scan 1 month after ablation. **C** CT scan 6 months after ablation. **D** CT scan 12 months after ablation. The red arrow indicates the HAE lesion. HAE, hepatic alveolar echinococcosis; MWA, microwave ablation

**Fig. 3:**
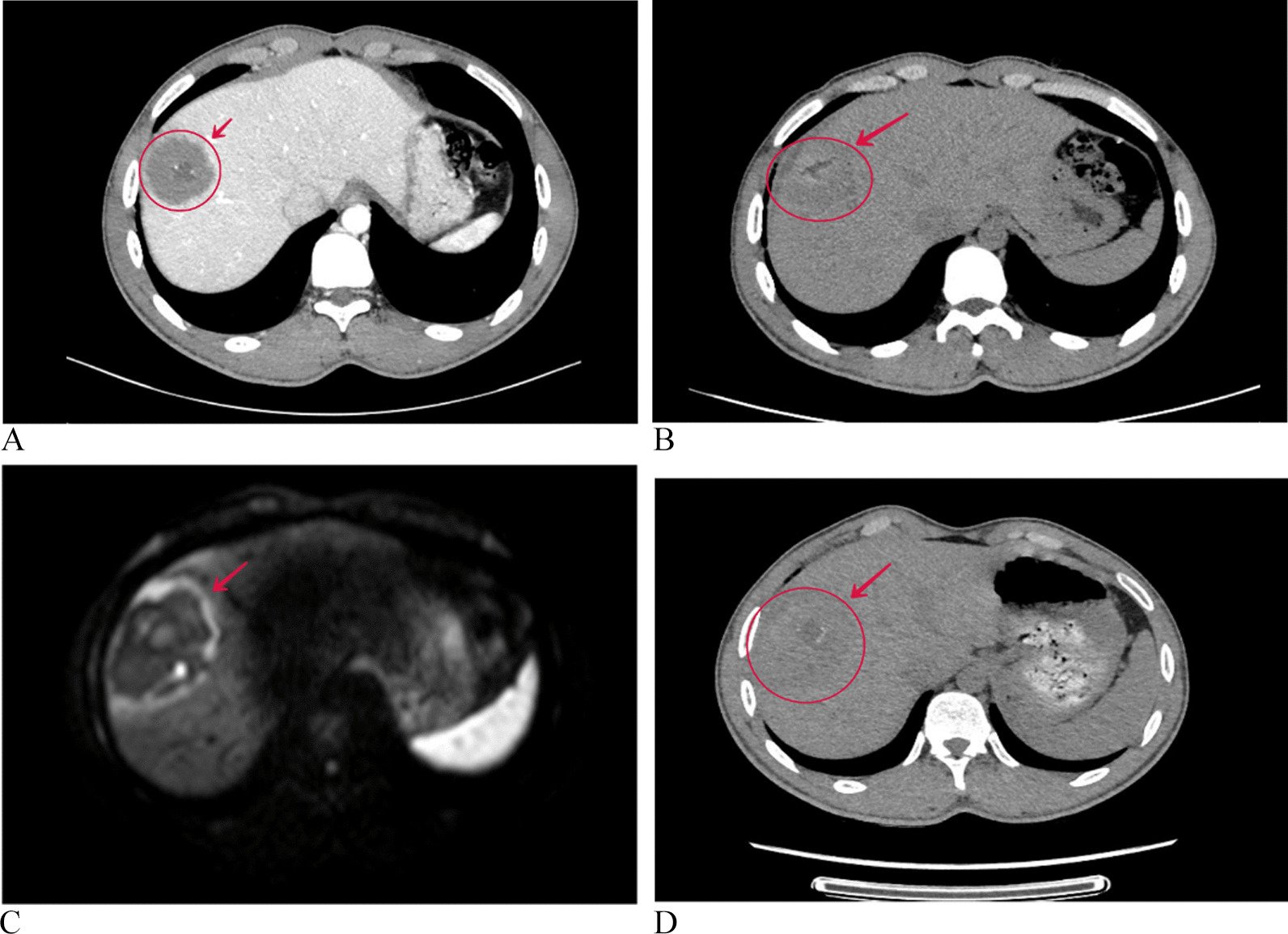
Images showing a lesion in an 18-year-old male patient with HAE recurrence before and after MWA treatment. **A** Enhanced CT was performed before the first ablation, and the lesion was marked by the red arrow. **B** CT scan on the first day after the first ablation, suggesting complete ablation. **C** At 17 months after MWA treatment, MRI-DWI suggested that the diffusion was limited around the lesions, suspected to be HAE recurrences. **D** CT scan on the first day after the second ablation suggesting complete ablation. HAE, hepatic alveolar echinococcosis; MWA, microwave ablation

The results of imaging examination within 24 h after operation showed that 100% (57/57) of the lesions were ablated completely (Fig. 2). After undergoing MWA, symptomatic patients were effectively improved before discharge. Additionally, as of January 31, 2021, the median follow-up time was 32 months (IQR 23–48.5). During the follow-up, the intrahepatic recurrence of HAE occurred in 13% (6/45) of patients, and the median time of recurrence was 22 months. Nobody had extrahepatic metastases and died. In the subsequent treatment of these recurrent patients, three patients continued to undergo MWA (Fig. 3) and two patients opted for radical hepatectomy. The remaining one patient refused microwave or radical resection and continued to take medication. As of the end of follow-up, there was no recurrence or further progression of the disease among the above 6 patients.

### The safety assessment of MWA

There were no serious complications or deaths during the procedure. The median operative time of 45 patients was 5.0 min (IQR 4.0–7.5). On the first day after the operation, the relevant laboratory indicators were monitored for each patient. ALB, RBC, HBG, and PLT were decreased after MWA (*p* < 0.001). At the same time, the postoperative indicators of ALT, TB, and DB were all higher than those before MWA (*p* < 0.001), and the paired-sample t tests showed that WBC were raised. In addition, there was no statistical difference between preoperative and postoperative indicators of PT, APTT, and INR (*p* > 0.05).

According to the Clavien–Dindo classification system, four grade 1 complications and one grade 2 complications were reported. All of them were minor complications (Clavien–Dindo grades I–II). Major complications (Clavien–Dindo grades III_a_–IV_b_) and mortality did not occur in this study. One patient with a lesion diameter of 4.26 cm presented with post-ablation syndrome (PAS), the hyperthermia caused by the release of inflammatory mediators due to tissue necrosis [14], and it only required supportive therapy. One patient with a lesion diameter of 3.12 cm and HBV developed postoperative hypoproteinemia without abdominal cavity effusion, which was treated with albumin supplementation. And three patients presented with asymptomatic pleural effusions and also did not require special treatment measures such as thoracic puncture or chest drainage. Their lesion diameter was 4.50 cm, 4.80 cm, 10.04 cm, respectively. Up to the time of discharge, the biochemical indexes of all patients showed normal or close to normal. Additionally, the median length of hospital stay after surgery was 5 days (IQR 3–6).

## Discussion

In order to verify the effectiveness and safety of MWA, the data of 45 patients were analyzed retrospectively. We found that MWA was characterized by short operative time, few postoperative complications, short postoperative hospital stay, and low recurrence rate. At the same time, compared to radical surgery, it was less traumatic for the patients, allowing for a higher quality of life. Finally, we believe that MWA is an effective and safe method for the treatment of HAE.

In our study, the complete ablation rate was 100%. Although the recurrence rate was 13% (6/45), we get the result is to be acceptable. Firstly, Salm et al. [15] showed that the recurrence rate of radical surgery for HAE was between 2 and 16%; and our post-operative recurrence rate is within this range. Secondly, in a study by Joliet et al. [16], it was noted that 2% (1/42) of patients with R0 resection had extrahepatic metastasis, and the probability of intrahepatic metastases in R1, R2 resected patients was 36% (5/14), and 66.7% (2/3), respectively. In addition, they pointed out that patients with non-R0 resection had a median recurrence time of 10 months. In contrast, the median time of recurrence was 22 months in our study, which was significantly longer than that in their study. Meanwhile, our patients did not present with extrahepatic metastases, and have a lower recurrence rate compared to non-radical resection.

In radical surgery, the common site of recurrence of HAE is at the edge of the surgical incision [17]. Similarly, our study also shows that the site of recurrence is commonly associated with the ablation margins. When MWA is used for liver cancer, recurrence also occurred near the ablation area [5, 18]. It’s believed to be due to the presence of an infiltrating area around the HAE lesion, which has characteristics of invasive growth similar to that of malignant tumors [19]. The main factor leading to the recurrence of HAE is that the marginal invasion zone is not completely inactivated. Since there is uncertainty regarding the extent of the infiltrative zone, in our experience, the ablation zone should cover at least 0.5 cm of liver parenchyma surrounding the lesion. We agree with the view that Albendazole (ABZ) should be applied for 2 years after any treatment of HAE [20].

In the study, we have shown that the efficacy of MWA is confirmed in lesions no larger than 5 cm in diameter. Since HAE is a benign disease, we also included patients with lesions larger than 5 cm in diameter, based on Wang et al.'s experience of treating giant hepatic hemangiomas with MWA [21]. But these lesions account for only 8.8% (5/57). Data on the effectiveness of MWA in large lesions are still insufficient. Although there were no recurrence or serious complications during the follow-up, other potential benefits of MWA were not evaluated in our study. Therefore, we still recommend that these patients choosing radical resection.

As reported by the National Institute for Health and Care Excellence, MWA is a safe treatment with “no major safety concerns” [22]. Meanwhile, two large, retrospective MWA studies showed major complication rates of 2.6% and 2.9% respectively [23, 24]. However, previous studies have shown that the incidence of postoperative complications in patients undergoing radical resection was 14%-40% [25, 26]. In an article exploring the surgical methods of HAE, Yang and colleagues [27] showed that the probability of minor complication and major complication after radical resection was 18.4% (16/87) and 9.2% (8/87) respectively, and there were two deaths. In the study by Joliet and colleagues [16], the probability of minor and major complications of the surgery was even higher, at 25% (15/59) and 9% (5/59), respectively. At the same time, a study has shown that the mortality rate of HAE patients undergoing radical surgery is 0–3.5% [28]. But, in our study, the rate of minor complications after MWA was 11.1% (5/45), and there were no serious complications and deaths. Additionally, these minor complications are usually self-limiting and do not require any further treatment. Although our patients had an increased post-procedure aminotransferase level, this only required supportive therapy. Andreano et al. [29] speculated that the total volume of ablation is associated with increased post-procedure aminotransferase levels. In our study, microwave ablation does not affect the patient's coagulation function. In addition, patients who underwent MWA had a faster postoperative recovery and a significantly shorter postoperative length of stay than patients undergoing radical surgery [27]. Therefore, we believe that microwave ablation is a safe method for the treatment of HAE.

In our experience, the relationship of the lesion to the hilar and intrahepatic vessels and bile ducts is an important key in the evaluation before MWA. With this as a starting point, we have developed the appropriate exclusion criteria. Among the patients, single (80.0%) and multiple lesions (20%) were involved, and lesions smaller than 3 cm, 3–5 cm, and > 5 cm were also included. The efficacy of MWA treatment could not be determined because the sample size of > 5 cm was too small to be included. However, for patients ≤ 5 cm, our study initially showed the effectiveness of its treatment.

In our study, one of the six patients who relapsed was P3N0M0, Kodama type 3; five were P1N0M0, Kodama type 1. According to Azizi et al. [10], Kodama type 1–3 is metabolically active. The recurrence in these six patients may be due to the failure to completely ablate the "infiltrative zone" around the lesion. However, the remaining metabolically active 44 lesions were ablated. Thus, our results suggest that MWA can be used in patients with P1N0M0 stage and Kodama type 1–3. Also, in conjunction with the study by Azizi et al. [10], we suggest that a follow-up can be adopted for patients with Kodama type 4–5. When the lesion tends to progress, aggressive surgical or MWA treatment is then promptly undertaken. Of course, we still need more studies to investigate the appropriate population for MWA to treat HAE.

The study with the largest number of cases and the longest follow-up period to evaluate the efficacy of MWA in the treatment of HAE. However, this study also has several limitations. First, the study was descriptive. So, we did not set up a control group. But we have compared the results of other researchers and confirmed the safety and efficacy of MWA to some extent. Secondly, the number of cases with a follow-up period of more than 5 years is still relatively insufficient. We need studies in this area to confirm the long-term recurrence rate of MWA. Third, we have only confirmed the effectiveness and safety of MWA for the treatment of lesions up to 5 cm in diameter. The performances of MWA in large lesions were not assessed in our study. Finally, this is a single-centered and retrospective work, which can easily lead to selection bias. Therefore, more researches are needed to verify our findings.

In conclusion, our results show that MWA is a safe and effective way to treat HAE. Meanwhile, it provides a new option and a new way of thinking about the treatment modality for patients with lesions ≤ 5 cm in diameter, P1N0M0, and Kodama type 1–3. And it has the possibility to replace radical surgery and drugs in the treatment of early HAE.

## Data Availability

The datasets generated and/or analyzed during the current study are not publicly available due patient privacy but are available from the corresponding author on reasonable request.

## Abbreviations

HAE: Hepatic alveolar echinococcosis
MWA: Microwave ablation
IQR: Interquartile ranges
ABZ: Albendazole
